# New approach to generating insights for aging research based on literature mining and knowledge integration

**DOI:** 10.1371/journal.pone.0183534

**Published:** 2017-08-17

**Authors:** Yeondae Kwon, Yukikazu Natori, Masaru Tanokura

**Affiliations:** Laboratory of Basic Science on Healthy Longevity, Department of Applied Biological Chemistry, Graduate School of Agricultural and Life Sciences, The University of Tokyo, Tokyo, Japan; University of Palermo, ITALY

## Abstract

The proportion of the elderly population in most countries worldwide is increasing dramatically. Therefore, social interest in the fields of health, longevity, and anti-aging has been increasing as well. However, the basic research results obtained from a reductionist approach in biology and a bioinformatic approach in genome science have limited usefulness for generating insights on future health, longevity, and anti-aging-related research on a case by case basis. We propose a new approach that uses our literature mining technique and bioinformatics, which lead to a better perspective on research trends by providing an expanded knowledge base to work from. We demonstrate that our approach provides useful information that deepens insights on future trends which differs from data obtained conventionally, and this methodology is already paving the way for a new field in aging-related research based on literature mining. One compelling example of this is how our new approach can be a useful tool in drug repositioning.

## Introduction

A lot of work has been done in the fields of health, longevity, and anti-aging since long-lived strains in *Caenorhabditis elegans* were first isolated [1]. In addition, the human genome project was completed in 2003 [2], and it has fundamentally altered methods of researching biological and physiological phenomena. It has also provided a vast amount of data to the research community. However, it is questionable whether this data is being used effectively in our quest to understand aging and longevity. Basic research results obtained from a reductionist approach in biology and a bioinformatic approach in genome science have limited usefulness for generating insights on future health, longevity, and anti-aging-related research on a case by case basis.

Bioinformatic analyses are used to provide information overviews, and extract valuable data by collecting and analyzing an extensive amount of information derived from various sources, including microarray [3, 4] and literature data [5]. The bioinformatic method has been incorporated into data analyses in biology, nutrition [6], and medical science [5] as well as aging research [7, 8], which explains why research terms such as translational bioinformatics [9, 10], health informatics [11, 12], and biomedical informatics [13, 14] have been defined and used on par with the newly coined term ‘bioinformatics’. These terms no longer describe fledgling disciplines but rather firmly established fields. However, the limits of reductionism still remain.

Deep learning as a machine-learning technique may have the potential to manage the limitations of reductionism on biological and physiological data and provides new insights on research trends [15]. This technique is capable of discovering intricate structures in high-dimensional data. Therefore, it is applicable to many domains of science. For example, deep learning was used to predict the activity of potential drug molecule [16] and the effects of mutations in non-coding DNA on gene expression and disease [17]. However, deep learning comprises layers of features that are not designed by human engineers. These layers are obtained using a general-purpose learning procedure of machine learning. For this reason, only the input provided by the researcher and the results gained from machine-learning are observed. Thus, this technique may sometimes provide unsatisfactory results because researchers usually pursue the question of “Why?” in the world of science. For example, even if aging-related genes are identified based on deep learning, the reasons why these aging-related genes were selected as genes related to aging will remain unclear.

In order to overcome the limitations of reductionism in this study, we implemented a new approach that incorporates bioinformatics into the literature mining method. Unlike results obtained solely from deep learning, this new approach provides an explanation as to why genes are selected as aging-related genes, and which genes should be focused on in future research. Furthermore, our approach is not confined to aging research and can provide new insights into various research fields, by the effective use of an expanded knowledge base.

## Results

It is indispensable to identify all aging-related genes involved in the aging phenomenon to comprehend the mechanisms of aging. In addition, overviewing the causal relationship between aging, disease, food, and lifestyle using these identified aging-related genes can lead to a better understanding of the mechanisms of aging, which will ultimately lead to the realization of healthy longevity. First, we identified aging-related genes by using our data mining techniques that identify the latest aging-related genes efficiently, effectively and comprehensively. Next, in order to verify the identified aging-related genes, we made a comparison to other aging-related databases. Then, in order to verify the usefulness of our new approach, we made a comparison between knowledge obtained via our new approach and data obtained via wet experiments. Finally, we demonstrated that our approach leads to a better perspective on research trends by providing an expanded knowledge base to work from. It can be observed that this methodology is already paving the way for a new field based on literature mining in aging-related research.

### Identifying aging-related genes

Aging is characterized by a progressive loss of physiological integrity, leading to impaired function and increased susceptibility to death. Recently, aging hallmarks, which are generally considered to contribute to the aging process, have been proposed [18, 19]. Using our efficient literature mining technique [20], we identified candidate aging-related genes that may be connected to these aging hallmarks.

First, we identified 45 aging-related terms (including ‘aging’, ‘senescence’ and ‘longevity) that occurred frequently in PubMed abstracts about aging (Table 1). Then, we identified 4,227 aging-related genes based on the hypothesis that a gene is an aging-related gene if it co-occurs with at least one of our pre-selected aging-related terms in a single sentence (S1 Table). A higher degree of accuracy is guaranteed by obtaining results based on co-occurrences within single sentences.

**Table 1:** Forty-five aging-related terms.

Aging-related terms	Number of aging-related genes based on single sentence co-occurrence	Number of aging-related genes based on abstract co-occurrence
AGE-ASSOCIATED	383	1,032
AGE-CORRELATED	5	14
AGE-DEPENDENCE	13	42
AGE-DEPENDENT	949	2,166
AGED-OBESE	4	8
AGE-INDUCED	139	333
AGEING	615	1,629
AGEING-ASSOCIATED	21	51
AGEING-DEPENDENT	4	9
AGEING-INDUCED	10	41
AGEING-LIKE	9	13
AGEING-RELATED	27	118
AGEING-SUPPRESSOR	0	0
AGE-MATCHED	1,533	3,106
AGE-RELATED	1,295	3,229
AGE-SENSITIVE	6	46
AGE-SPECIFIC	191	693
AGING	1,885	4,317
AGING-ASSOCIATED	105	353
AGING-DEPENDENT	24	78
AGING-INDUCED	39	151
AGING-LIKE	20	68
AGING-RELATED	185	566
AGING-SUPPRESSOR	4	21
ANTI-AGEING	17	103
ANTI-AGING	87	365
HEALTH-RELATED	151	789
HEALTHSPAN	17	94
HEALTHSPANS	0	0
LIFE EXTENSION	33	82
LIFE-EXTENDING	8	36
LIFESPAN	832	2,190
LIFE-SPAN	115	362
LIFESPAN:	3	6
LIFESPANS	55	217
LIFE-SPANS	7	33
LONGEVITY	626	1,664
LONG-LIVED	485	1,360
LONG-LIVING	30	107
SENESCENCE	1,597	3,147
SENESCENCE-ACCELERATED	45	258
SENESCENCE-ASSOCIATED	322	841
SENESCENCE-LIKE	103	244
SENESCENCE-RELATED	63	169
SENESCENT	711	1,466

However, certain genes with the potential to be classified as aging-related genes will not be selected based on this criterion. Hence, by changing the criteria to co-occurrences in ‘the same abstract’, 7,416 aging-related genes were identified from literature collections (S2 Table). Are the 3,189 genes that were not selected on the ‘single sentence’ criterion true aging-related genes? The answer to this question will provide clues to the most useful criteria to use when identifying aging-related genes. In statistics, a confidence interval can be used to describe how reliable survey results are. For this study, a 95% confidence interval, which reflects a significance level of 0.05, was applied used by using the following formula:

$$R-1.96\sqrt{\frac{R\left(1-R\right)}{n}}<p<R+1.96\sqrt{\frac{R\left(1-R\right)}{n}}$$ (1)

We randomly selected 127 out of 3,189 genes, and we determined that 115 out of 127 are aging related genes. We used *n* = 127, and $R=\frac{115}{127}=0.906$, and obtained a confidence interval of 85.5% to 95.6%. Similarly, we randomly chose 4 more different sets of 127 genes, and we obtained the following confidence intervals: 82.6% to 93.8%, 87.4% to 96.8%, 88.5% to 97.4%, 90.5% to 98.5%. We have decided whether the genes that we selected randomly are true aging-related genes, by examining the relationships between genes and aging-related terms in abstract texts. When this technique used, some aging-related genes may appear as false positives. Consequently, the lower limit of the confidence interval is likely to be greater than 82.6% when considering the entire paper rather than just the abstract. On this basis, we decided that it would be best to use ‘the same abstract’ as the criterion for identifying aging-related genes.

In order to obtain a more comprehensive list of aging-related genes, we not only used literature mining, but we also used the pathway hypothesis, which states that a gene is an aging-related gene if it occurs in the same pathway as a pre-established aging-related gene. This hypothesis is based on the assumption that genes involved in the same biological phenomenon belong to the same pathway. We identified 1,339 additional aging-related candidate genes based on the pathway hypothesis. We also did an in-depth investigation of one of these additional candidate genes, which is the TNF receptor associated factor 5 (TRAF5) gene. It occurs in the NF-kappa B signaling pathway (hsa04064) of the poly (ADP-ribose) polymerase 1 (PARP1) gene, which was identified as an aging-related gene by our literature mining technique. The NF-kappa B activation has been reported as a hallmark of the aging process [21]. In addition, it has been reported that TRAF5 regulates the IL-6R signaling needed for Th17 development [22], and the Th17/Treg balance is disturbed during aging [23]. These results suggest the following: one is that TRAF5 is an aging-related gene, and the other is that extracting genes that occur in the same pathway as aging-related genes is a valid method for identifying the highest number of aging-related genes.

However, if pre-selected aging-related genes identified by literature mining include false positives, aging-related genes identified based on the pre-selected aging-related genes are likely to include false positives as well. Therefore, the research strategy may be determined on the basis of the researcher’s aims. Some researchers may favor the highest number of results whereas others will favor the most accurate results. For example, the following strategy can be adopted to reduce false positives and accurately select aging-related genes: identify reliable aging-related genes by intentionally using restricted criteria and then select genes located on the same pathway as the pre-selected aging-related genes to identify the highest number of aging-related genes.

### Verifying aging-related genes

In order to verify identified aging-related genes, the data from four public databases were used: GenAge [24], AgeFactDB [25], Digital Ageing Atlas [26] (hereafter called ‘Atlas’), AGEMAP [27]. In this study, because we focused on protein-coding genes obtained from human gene data, non-human and non-protein-coding genes in these databases were removed as verification data. For example, a non-coding RNA gene telomerase RNA component (TERC) was excluded from GenAge, and 304 genes out of 305 genes in GenAge were used. Additionally, 6,742 human homologous genes corresponding to 8,932 mouse genes in AGEMAP were used.

We checked whether the identified aging-related genes covered all of the genes in GenAge, because GenAge offers benchmark data for aging-related genes. By incorporating pathway information, the coverage of the genes in GenAge was increased from 90.1% (274 genes) to 94.4% (287 genes) for co-occurrences in a single sentence and from 96.1% (292 genes) to 99.0% (301 genes) for co-occurrences in the same abstract (see the second column from the right in Table 2). Incidentally, as shown in Table 2, AgeFactDB covered 94.1% (286 genes) of the genes in GenAge, which was similar to the percentage covered by our database, whereas Atlas and AGEMAP covered only 23.4% (71 genes) and 55.3% (168 genes), respectively.

**Table 2:** Comparison of four public databases.

Aging database	Number of aging genes	GenAge Coverage^†^	Sirtuin genes
Single sentence co-occurrence (Including gene on the same pathway	4,227 6,140	274 (90.1%) 287 (94.4%)	SIRT1-SIRT7
Abstract co-occurrences (Including gene on the same pathway)	7,416 8,755	292 (96.1%) 301 (99.0%)	SIRT1-SIRT7
GenAge	304	-	SIRT1, SIRT3, SIRT6, SIRT7
AgeFactDB	856	286 (94.1%)	SIRT1-SIRT3, SIRT6, SIRT7
Atlas	2,599	71 (23.4%)	SIRT1, SIRT5
AGEMAP	6,742	168 (55.3%)	SIRT1, SIRT2, SIRT7

^†^Number of genes in common with the genes in GenAge.

In addition, we compared the coverage of the candidate aging-related genes identified by our method, and those present in other aging databases. The sirtuin genes SIRT1—SIRT7 in mammals, which are also known as longevity-related genes, have received significant attention for their regulatory role in metabolism and aging [28–30]. It has been reported that these longevity-related genes mediate both the anti-aging effect and lifespan extension by way of calorie restriction [31]. We found that candidate aging-related genes identified by our literature mining approach contained all of the sirtuin genes, whereas the other databases did not despite being published after all the sirtuin genes had been reported (see the rightmost column in Table 2). These results suggest that our literature mining approach is effective at rapidly identifying the latest aging-related genes, even when allowing for false positives.

### Classification and overview generate insights

The purpose of knowledge integration via heatmaps [32] is to understand the relationship between aging-related genes and aging-related causes and/or aging-related diseases. Heatmaps are a tool for obtaining classifications and overviews, and they help predict the future of aging-related research. For this study, heatmaps were produced based on the Dice score, which is used to measure the association strength of two words. Dice is defined as follows:

$$Dice\left(x;y\right)=\frac{2\cdot f\left(x;y\right)}{f(x)\cdot f\left(y\right)}$$ (2)

*f*(*x*;*y*) being the frequency of co-occurrence of *x* and *y*, and *f*(*x*) and *f*(*y*) are the frequency of occurrence of *x* and *y* anywhere in the abstract. If *x* and *y* tend to occur in conjunction, their Dice score will be high. The Dice score ranges from 0 to 1, where 1 indicates a perfect overlap.

**Fig 1:**
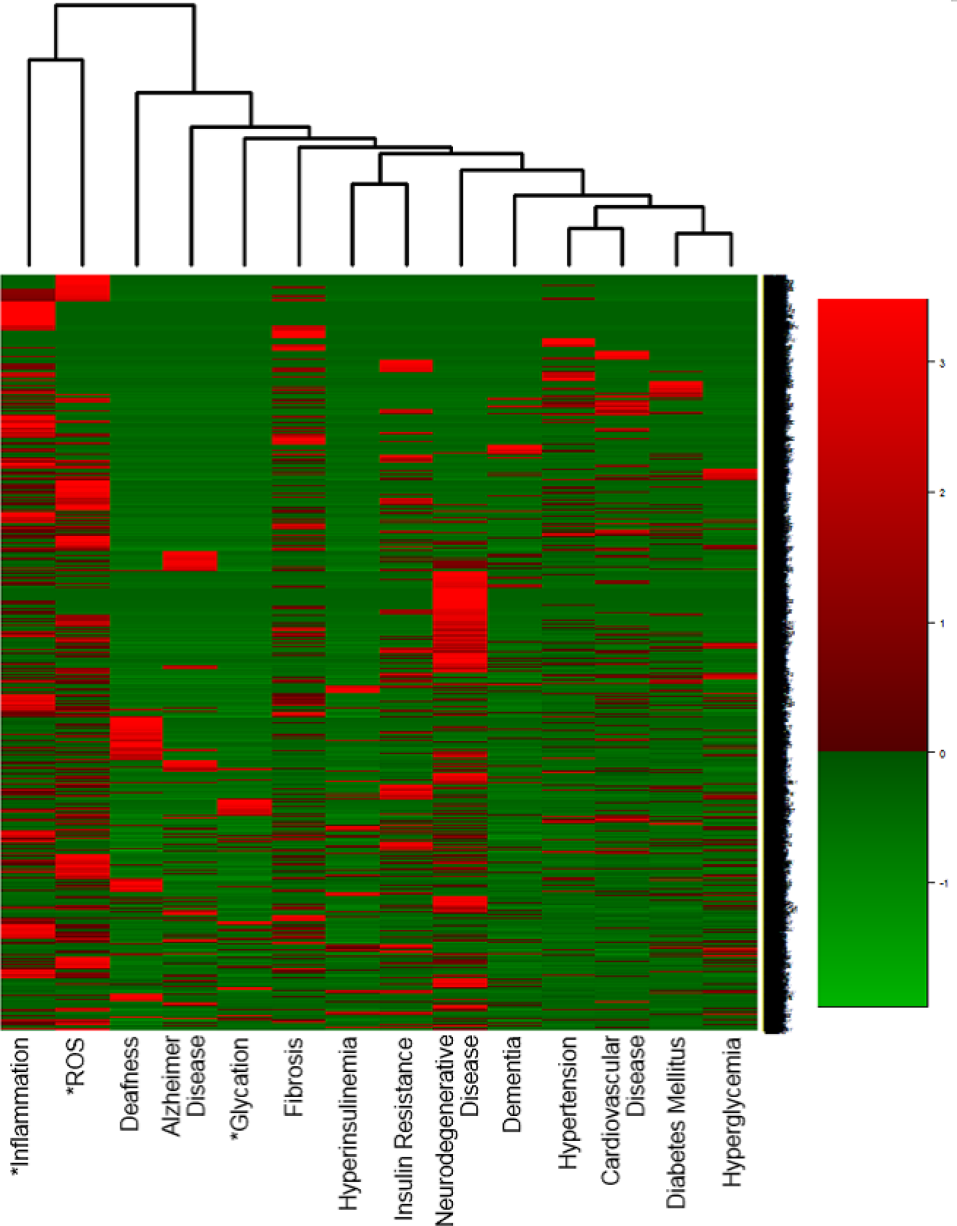
Heatmap providing an overview of 6,551 aging-related genes. Heatmaps provide overviews of the relationships between aging-related genes and aging-related causes and/or aging-related diseases. Aging-related causes are marked with an asterisk. Three aging-related causes and 11 aging-related diseases were selected for this paper. Out of our 7,416 aging related genes, 6,551 genes co-occurred with at least one of the 14 terms we selected. The vertical axis consists of all 6,551 genes.

The heatmap in Fig 1 was produced for 7,416 aging-related genes identified by literature mining, 3 aging-related causes and 11 aging-related diseases. Aging-related causes and diseases can be selected based on specific research objectives, and in this case 14 terms were selected. 865 out of 7,416 aging-related candidate genes did not co-occur with any of the14 terms on the horizontal axis. A possible explanation for this is that though these genes did not co-occur with our 14 selected terms, they may have co-occurred with other aging-related diseases. There were only 128 genes out of 7,416 that co-occurred with all 14 terms (S3 Table). Although there is some fluctuation, overall most of these genes show a gradually increasing number of co-occurrences with aging-related terms in PubMed abstracts, and they have been studied from various standpoints such as pathways and disease. Therefore, these genes may be a key to understanding the mechanisms involved in aging.

**Fig 2:**
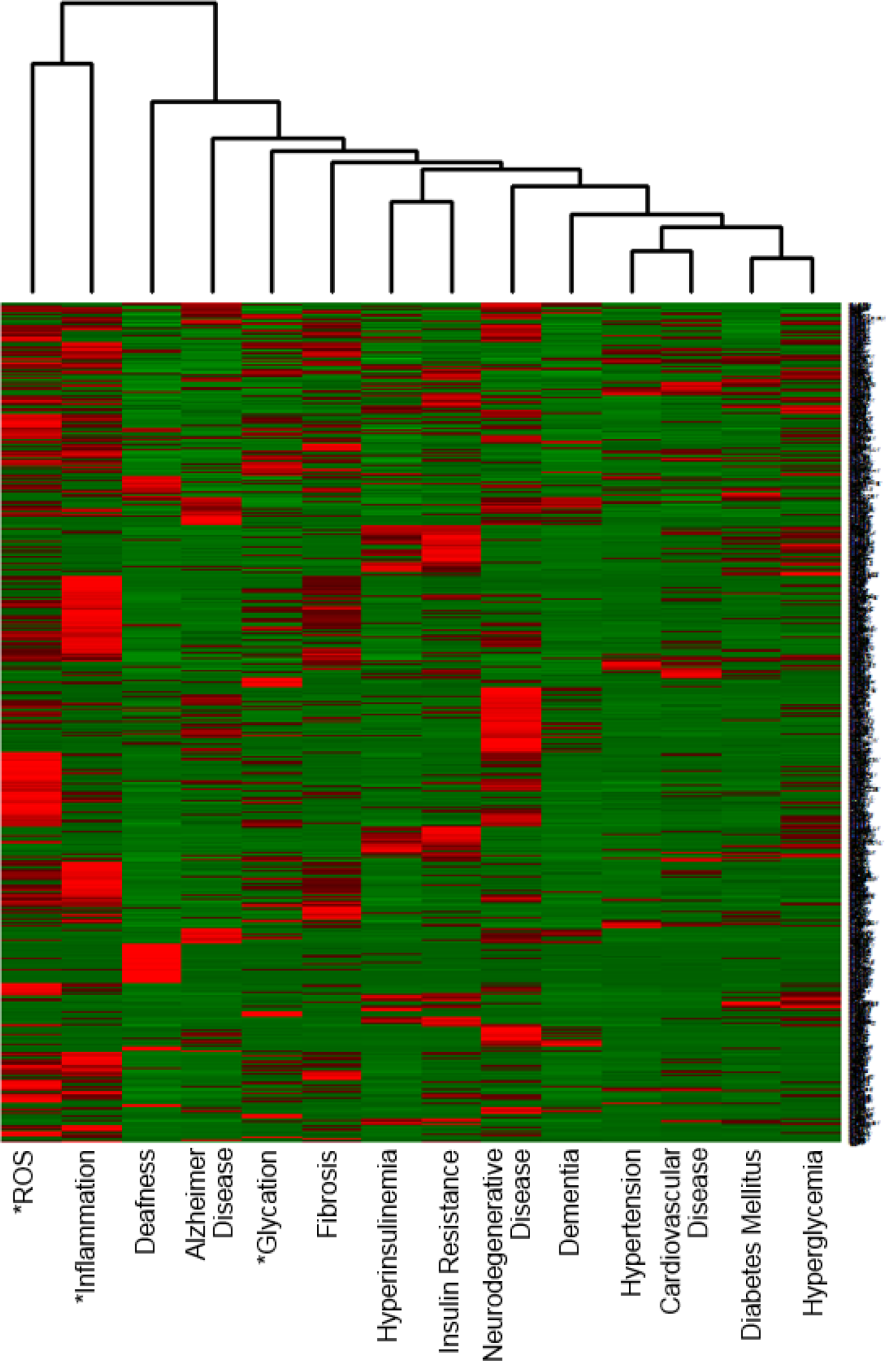
Heatmap providing an overview of the top 1,000 genes. The vertical axis consists of the top 1,000 genes out of 6,551 aging-related genes. Although any number of aging-related genes can be placed on the vertical axis, in this case, 1,000 were chosen. These genes were selected on the basis of their co-occurrences with the aging-related causes and/or diseases on the horizontal axis in the highest number of publications.

The vertical axis of Fig 1 consists of all 6,551 genes, which co-occurred with at least one of 14 terms, while Fig 2 consists of the top 1,000 genes out of 6,551 aging-related genes. Any number of aging-related genes can be placed on the vertical axis. In Fig 2, 1,000 were chosen due to their co-occurrences in the highest number of publications. However, the higher the number of aging-related genes chosen, the more comprehensive the overview. Interestingly, by comparing Figs 1 and 2, it can be seen that Reactive oxygen species (ROS) and inflammation share most of the same aging-related genes, whereas glycation doesn’t. Out of 6,551 genes, there are only 915 that are involved in glycation, and 590 out of 915 genes are listed in the top 1,000 aging-related genes. As far as the 5,551 genes besides the top 1,000 are concerned, only 325 (0.06%) are involved in glycation. This may suggest that glycation has not been as thoroughly studied as it should be in relation to aging. This conclusion may become a guideline for future research.

ROS and inflammation are in close proximity to deafness on the heatmap. Consequently, it can be inferred that ROS [33] and inflammation [34] are deeply related to cause of deafness. In addition, it is likely that glycation shares more of the same aging-related genes with AD and Fibrosis than it does with other diseases, due to its proximity to them. This finding suggests that reducing glycation is an important method for preventing or delaying AD and Fibrosis.

As far as aging-related diseases are concerned, the close proximity between deafness and AD on the heatmap indicates that Deafness and AD are closely related. Incidentally, it has been established that there is a significant link between hearing loss and the development of dementia [35–38]. In addition, the heatmap shows that the following pairs are in close proximity: Hyperinsulinemia and Insulin resistance, Hypertension and Cardiovascular disease, and Diabetes mellitus and Hyperglycemia. Therefore, it is likely that these pairs share the same aging-related genes. Moreover, brain-related diseases, such as Neurogenerative disease and Dementia, are closer to the four diseases on the far right-hand side: Hypertension [39, 40], Cardiovascular disease [41–43], Diabetes mellitus [44–46], Hyperglycemia [47, 48]. This is a demonstration of how our approach can provide overviews of scientific literature to date, and reaffirm scientific results that are understood in the science world.

**Fig 3:**
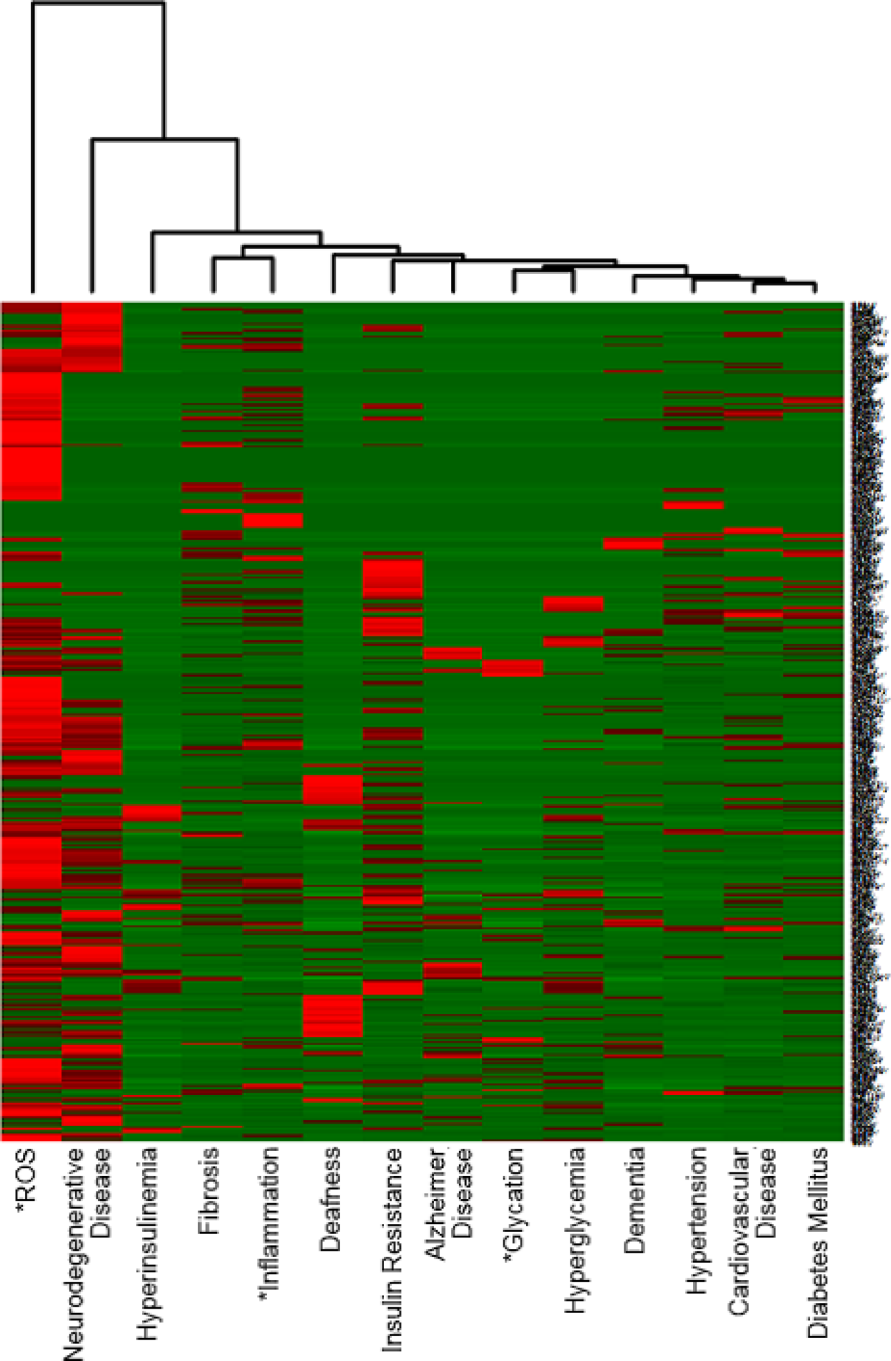
Heatmap providing an overview of aging-related genes that are related to mitochondria. Out of our 7,416 aging related genes, only 512 genes are related to mitochondria. The vertical axis consists of the 474 mitochondria-related genes that co-occurred with at least one of 14 terms we selected.

Mitochondria play a central role in energy production, and their dysfunction is thought to be involved in aging, Diabetes mellitus, Neurodegenerative diseases and Cancer [49–51]. The heatmap in Fig 3 was produced for the 474 mitochondria-related genes that co-occurred with at least one of the14 terms we selected. MitoCarta2.0 [52], which is a public database, was used to acquire information about mitochondria proteins. This heatmap shows that aging-related genes that are associated with mitochondria co-occur more frequently with ROS than inflammation, which is considered by many to be the most common cause of aging. This result suggests that the progression of aging in mitochondria might be mainly caused by ROS. The mitochondrial theory of aging postulates that reactive oxygen species (ROS) generated inside mitochondria damage key mitochondrial components, including mitochondria DNA (mtDNA) [53]. Such damage accumulates with time and major phenotypes associated with aging are caused by mitochondrial dysfunction [53, 54].

The reason there is a difference between Figs 1, 2, and 3 is follows: Fig 1 has all aging-related candidate genes, Fig 2 has the top 1,000 genes on the vertical axis, and Fig 3 has been narrowed down to aging-related genes that are connected to Mitochondria. The perspective offered by the heatmap depends on the information in both the vertical and the horizontal axis. Therefore, one should keep in mind that the heatmaps provided in this paper offer only a limited number of perspectives out of the many potential overviews possible. Consequently, the knowledge provided by these heatmaps should be considered a working hypothesis rather than completely factual data.

**Fig 4:**
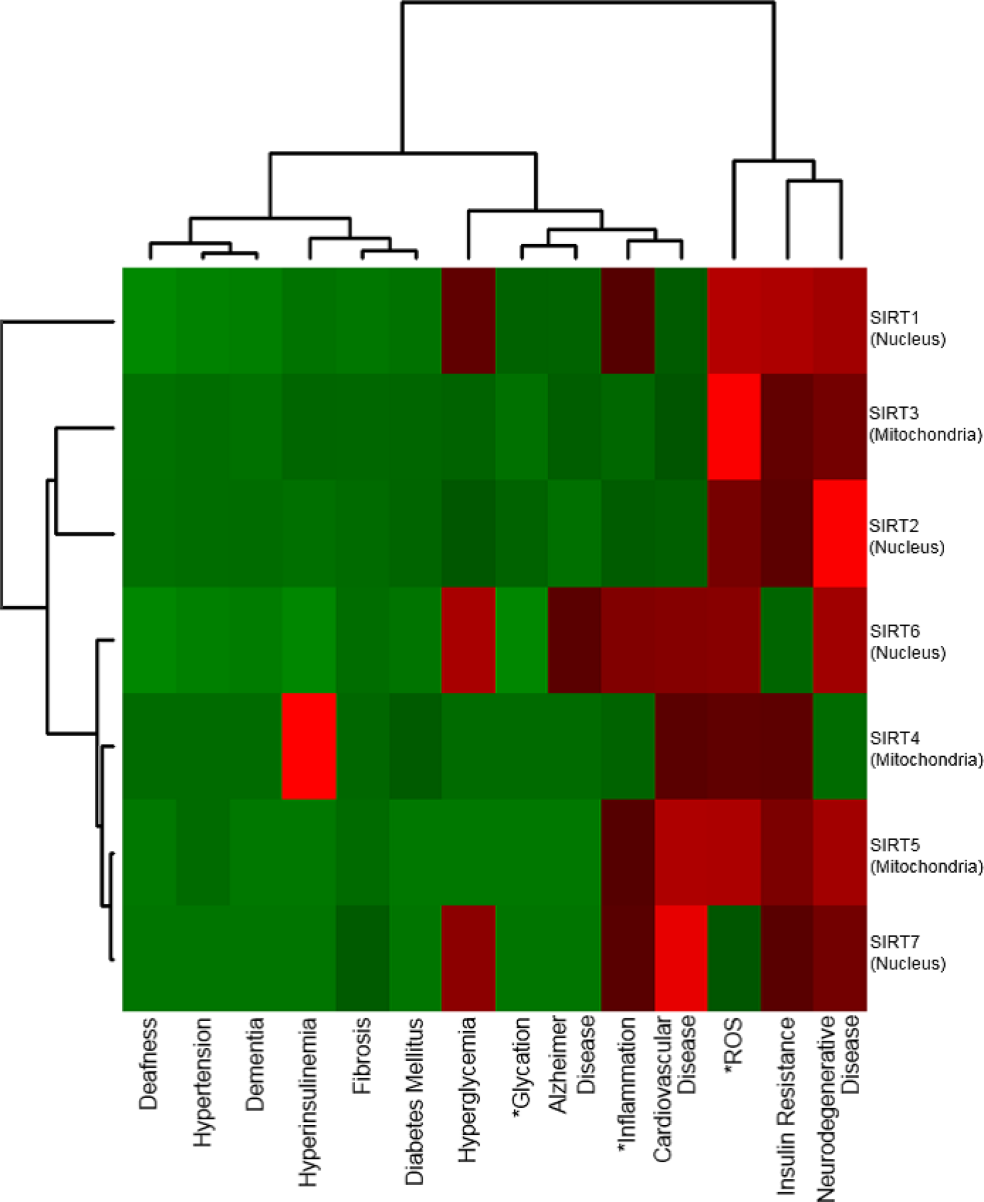
Heatmap providing an overview of sirtuin genes. The vertical axis consists of sirtuin genes SIRT1-SIRT7. This heatmap shows that SIRT2, SIRT3, and SIRT5, SIRT7, form two separate clusters. SIRT3 and SIRT5 affect the mitochondria, while SIRT 2 and SIRT7 affect the nucleus. Therefore, we can infer that these two clusters may work interchangeably.

Fig 4 summarizes the results for the longevity genes SIRT1-SIRT7. The gene locations are written in parenthesis on the vertical axis. From this heatmap, it can be seen that SIRT2, SIRT3, and SIRT5, SIRT7, form two separate clusters. We can infer that the genes in each cluster may have similar functions. However, while SIRT3 and SIRT5 affect the mitochondria, SIRT 2 and SIRT7 affect the nucleus. Therefore, these two clusters may work interchangeably. It can be seen that SIRT4 is more closely related to Hyperinsulinemia than other sirtuin genes, and Hyperglycemia has been well researched in relation to SIRT1, SIRT6, and SIRT7. In addition, these sirtuin genes are all located in the nucleus, and are related to the same diseases including Neurogenerative diseases, Insulin resistance, and Cardiovascular disease. It can also be predicted that these three genes are involved in similar mechanisms of aging.

Surprisingly, no publications with co-occurrences between deafness and sirtuin genes were found. In contrast, we found that ‘age-related hearing loss’ co-occurred with 118 genes including SIRT1,2,3,6, and 7. The first result was obtained by using 'deafness' as the keyword disease name for literature mining, while the second was obtained using 'age-related hearing loss'. Considering these results, it is clear that selecting disease names and/or causes precisely is important in the prediction of significant genes for future aging-related research.

By using our literature mining approach, we can firstly identify the genes of interest quickly, including the latest genes, and then overview the relationships between aging-related genes and aging-related causes and/or aging-related diseases. In addition, our new approach can provide both an overview and a highly-detailed view, which is useful in gaining new insights. Consequently, we can obtain useful information that deepens insights on future trends, which differs from data obtained conventionally.

## Discussion

### Gene dictionary

Generally, a gene has a primary gene symbol and a number of aliases. The publications where the gene occurs are mined for both primary gene symbols and the corresponding aliases. However, this can lead to false positives for the following two reasons: a primary gene symbol can be used as an alias for other genes, and (in such cases) the primary gene symbol was deleted as the alias of other genes, and the same aliases can also be used for other genes (which requires the identification of the genes that correspond to the alias). This problem must be considered. Measures were taken to mitigate this problem in our study. However, they are not addressed in this paper.

### Identifying aging-related terms

**Fig 5:**
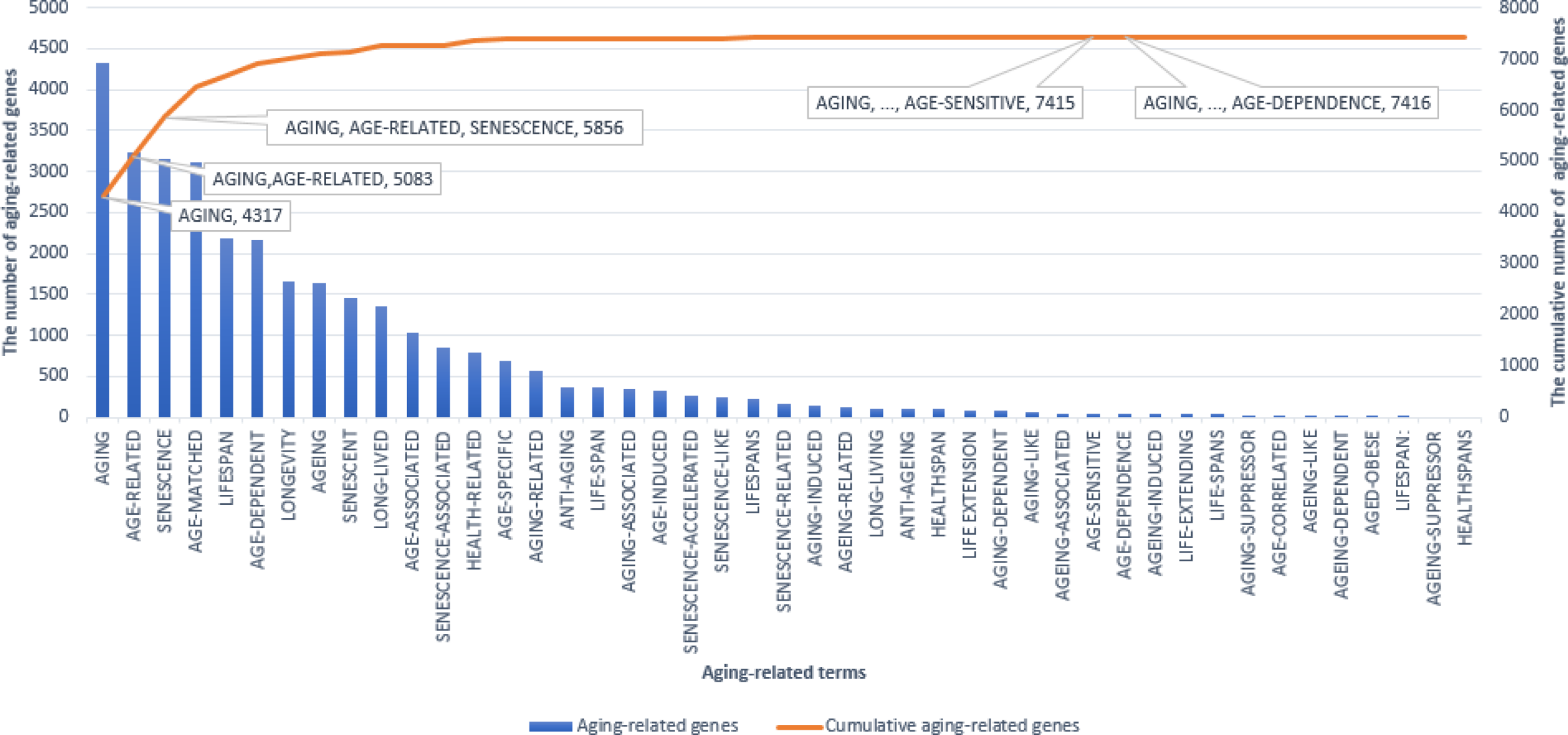
Histogram representing the cumulative number of aging-related genes. The blue bars show the number of aging-related genes for each individual term and the orange line represents the cumulative number of aging-related genes identified by the terms progressively. For example, 5,083 aging-related genes were identified by using the terms “aging” and “age-related”, and when the term ‘senescence’ is added, the total comes to 5856.

Aging research has been done for various research purposes, such as clarifying the cause of disease, preventing aging, and realizing health longevity. In order to include so many aspects of aging research, we selected a wide range of aging-related terms, which are involved in aging, anti-aging, and health longevity as candidate terms, and which also occurred frequently in PubMed abstracts about aging. In this paper, we regard a gene as an aging-related gene if it co-occurs with at least one of these aging-related terms in a single sentence or the same abstract. In order to reduce false positives, we considered a method that can cover more aging-related genes using fewer aging-related terms. By viewing a histogram (Fig 5), we were able to see that 45 aging-related terms were both necessary and sufficient to identify all aging-related genes. In this graph, we can see that, by using the terms from ‘aging’ to ‘age-dependence’ exclusively, the cumulative number of aging-related genes that correspond to our 45 aging-related terms is 7,416. This indicates that our 45 aging-related terms are sufficient to identify all of the aging-related genes needed to meet our research objectives.

**Fig 6:**
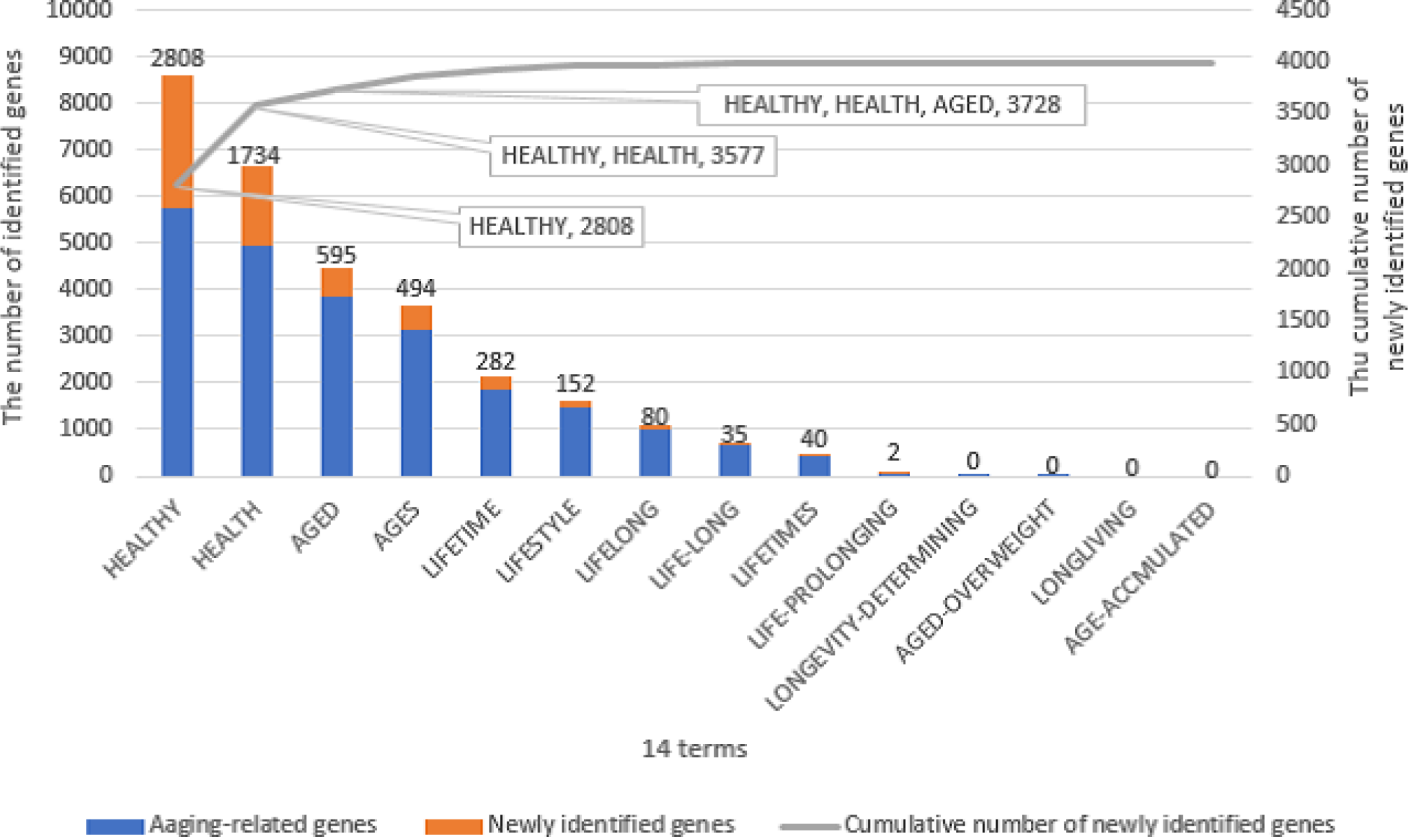
Histogram of the 14 terms that were discarded. The blue bars show the number of genes that are included in the group of 7,416 aging-related genes identified by using 45 aging-related terms. The orange bars show the number of newly identified genes for each term, and the gray line represents the cumulative number of newly identified genes by the terms progressively.

In addition to the 45 aging-related terms chosen, we originally listed another 14 terms that were eventually discarded (Fig 6). We questioned whether they should be considered aging-related terms. We identified 8,582-related genes by using only ‘healthy’ as shown in Fig 6. 2,808 of them did not co-occur with any of the 45 aging-related terms chosen, and 5,774 of them had already been identified in the group of 7,416 aging-related genes. This means that every one of those 5,774 genes co-occurred with at least one of the 45 aging-related terms, aside from healthy, in the same abstract. Incidentally, 1,642 out of 7,416 aging-related genes were not identified by using ‘healthy’. This means that though the term ‘healthy’ can identify a wide range of genes, is too versatile to identify specific genes. This study is aimed at clarifying the mechanisms of aging and making healthy longevity attainable. While the 5,774 genes are likely to be involved in both aging and healthy longevity, the additional 2,808 genes widen the scope beyond that. We have decided not to use ‘healthy’ as an aging-related term, or consider the 2,808 new genes, which were identified by using ‘healthy’, as healthy longevity genes related to aging for this reason. If the scope of the research requires it, these genes can be included. There are various types of aging-related research and we should choose aging-related terms according to their purpose. In our opinion, this potential flexibility is one powerful advantage of text mining. We found that the higher number of aging-related terms used, the higher the number of false positives present in the newly discovered aging-related genes. Therefore, we listed 45 aging-related terms that satisfy our requirements for avoiding both false positives and false negatives aging-related genes.

### Identifying aging-related genes

In this study, we proposed two hypotheses for identifying aging-related genes. One is that a gene is an aging-related gene if it co-occurs with at least one of our pre-selected aging-related terms in a single sentence or the same abstract. Another is that a gene is an aging-related gene if it occurs in the same pathway as a pre-established aging-related gene. In order to guarantee the accuracy of identified aging-related genes we should choose the ‘single sentence’ criterion. Conversely, in order to guarantee quantity, we should choose to use the pathway hypothesis. In this study, in order to guarantee both sufficient quantity and quality, we chose to use the 'same abstract' criterion, and identified 7, 416 aging-related genes, which consist of 4,227 aging related gene with co-occurrences in the same sentences, and 3,189 with co-occurrences in the same abstracts.

In Contrast, the classification of false positives depends on the focus of the research. Therefore, we determined that certain genes, which were not in alignment with our research objectives, were false positives. The tally of false positives is dependent on the criterion used to identify the aging-related genes. We randomly selected 845 out of the 4,227 genes (20%) and 638 out of the 3,189 genes (20%) in order to check for false positives, and we found that 41 and 53 genes were not aging-related genes, respectively. It is better to avoid giving a specific percentage, as the definition of false positives depends on the focus of the research. Our research gives false positives to some extent. Even when attempting to guarantee quality by using the single sentence criterion, false positives occur. Most of these false positives are due to gene symbol occurrences, because the gene symbol has the same spelling as acronyms, which symbolize other gene names or phrases unrelated to genes. In terms of reducing false positives, our study was successful to a certain extent. However, this will be the focus of our further research as more work is needed in this area.

In any case, one of the goals of this study is to propose genes that will be of interest to experimental researchers in the future. Whether genes can suppress or promote disease, it is important to recognize the fact that both types of genes have been attracting attention as research subjects. The advantage of text mining is that it can be used to gain high quality coverage. In addition, it is possible to narrow the focus down to genes of interest that match specific research objectives by using keywords, which are related to research objectives with a particular focus.

### Comparison to other aging databases

Recently, several databases developed for aging-related genes have become widely accessible. In order to verify our identified aging-related genes, we compared them to aging-related genes in the following four databases: GenAge, AgeFactDB, Atlas, AgeMap. All the genes related to aging must be identified to understand the molecular mechanisms of aging and related diseases. The databases mentioned above are manually curated and provide information on high-quality aging-related genes, although they do not cover all of the genes that are associated with the aging process. However, because our approach is based on the scientific journal literature published to date, our database covers potential aging-related genes published so far.

### Providing new insights

We investigated how many of our aging-related genes were used as target genes for known drugs, in order to demonstrate that our approach is an efficient tool for discovering new therapeutic indications for existing drugs. We found that 522 out of 7,146 aging-related genes that we identified using our literature mining approach are used as the target genes for known drugs. These genes can be used as a primary filter for discovering new uses for approved drugs. Metformin targets AMP-activated protein kinase (AMPK), and has been used to treat diabetes since the 40’s [55]. It is currently in clinical trials as an anti-aging drug [56]. Genes known as AMPK are PRKAA1 and PRKAA2, and they are aging-related genes. Moreover, 38 out of 128 genes that co-occurred with 14 terms that are deeply related to aging are used as the target genes for known drugs. For example, the TLR2 gene is targeted by Erlotinib (DB00045), which is used to prevent Lyme Disease. This gene not only exists on pathways involved in aging and longevity such as MAPK signaling (hsa04010) and PI3K-Akt signaling (hsa04151), but it also exists on the following signaling pathways: leishmaniasis (hsa05140), malaria (hsa05144), toxoplasmosis (hsa05145), amoebiasis (hsa05146), tuberculosis (hsa05152), hepatitis B (hsa05161), measles (hsa05162), inflammatory bowel disease (hsa05321), rheumatoid arthritis (hsa05323), etc. TRL2 is clearly related to signaling pathways involved in aging and infection. The use of Erlotinib can be considered a possible treatment for aging-related diseases and infection-related diseases in the absence of contraindications related to side effects. For this reason, our approach is considered highly effective as a springboard to new discoveries that can lead to new therapeutic indications for existing drugs, commonly known as drug repositioning.

In this study, to overcome the limitations of reductionism, we implemented a new approach that incorporates bioinformatics in the literature mining method. The results obtained based on our approach can explain why genes are selected as aging-related genes and which genes should be focused on in future research. Furthermore, our approach can also provide new insights into various research fields, by the effective use of an expanded knowledge base. It would be also beneficial to collaborate with wet-lab researchers, in order to help them expedite the selection process for future research, and to fine tune our approach on the basis of their feedback.

## Materials and methods

Our approach consists of 3 parts: creating term dictionaries and literature databases, identifying aging-related genes, and classifying and providing an overview of the integrated information. The details of each step are described below.

### Creation of term dictionaries and a literature database

In this step, 3 term dictionaries used as keywords for literature mining are created and a document database is constructed.

#### Gene dictionary

The human gene data from the FTP site of the National Center for Biotechnology Information (NCBI) (ftp://ftp.ncbi.nlm.gov/gene/DATA/) for July 2016 were downloaded. First, gene IDs, symbols, synonyms, and gene name fields from the data were selected. Next, the genes that were indicated as protein coding in the ‘Type’ field were selected. Then, when a primary gene symbol could be used as an alias for other genes, the primary gene symbol was deleted as the alias of other genes. The resulting gene dictionary contained a total of 68,260 entries, including gene synonyms (S4 Table).

#### Disease dictionary

The disease terms from the Comparative Toxicogenomics Database (CTD) [57], which provides curated disease names for July 2016, were used. First, primary disease names that had MeSH IDs or OMIM IDs were selected. Next, disease synonyms that were not specific to a single disease and/or have the same spellings as general words were deleted. Then, the diseases terms that were indicated as animal diseases in the SlimMapping field were deleted. The resulting disease dictionary contained a total of 75,756 entries, including disease synonyms.

#### Aging-related term dictionary

To collect aging-related terms, GenAge, which is an aging gene database that provides benchmark data for aging-related genes [24], was used. GenAge offers information on 305 aging-related genes and their PubMed IDs released in October 2015. Frequent terms relevant to aging and longevity in PubMed abstracts were extracted based on PubMed IDs, with the exclusion of general terms such as ‘is’, ‘a’ and ‘this’. Finally, 45 terms related to aging and longevity were listed (Table 1).

#### Literature database

To form the foundation of our literature database, MEDLINE/PubMed abstracts from NLM (ftp://ftp.ncbi.nlm.nih.gov/pubmed/) for June 2016 were downloaded and information from a total of 27,420,471 documents was obtained. Because the connections between genes, aging-related terms, and disease names were evaluated by using the aforementioned dictionaries and identifying frequently used terms in the Abstract Text fields, documents that did not contain these fields were eliminated. Finally, the PubMed IDs and Abstract Text fields were extracted from the remaining documents, which totaled 17,541,101.

### Identifying aging-related genes

To identify aging-related genes, the following hypothesis was applied: a gene is an aging-related gene if it co-occurs with aging-related terms in the same sentences. In this study, the co-occurrence frequency of gene symbols and aging-related terms in the literature was used as the evaluation criteria for relationships between aging-related terms and genes. The higher the number of publications in which the gene and aging-related terms co-occurred, the more likely that the gene was an aging-related gene. Specifically, the following procedures were used:

First, all of the gene symbol occurrences were extracted from our non-redundant PubMed abstracts by performing a keyword search. Because gene symbols are generally created from the acronyms of gene names, certain symbols, such as the gene IMPACT (imprinted and ancient gene protein homolog), have the same spellings as general words, and these symbols may produce false positives in keyword searches. Therefore, in addition to the keyword search, additional gene symbol occurrence checks were performed, which are referred to as neighbor searches [20]. Neighbor searches check whether any of the words that have been abbreviated to form a symbol, which can be called a neighbor search word set, appear near the symbol in a single sentence. If any word from the neighbor search word set is found in the same sentence as the corresponding gene symbol, the occurrence is considered to be positive. The gene occurrence table consists of Gene IDs, PubMed IDs, and the sentence numbers where the corresponding gene symbols appear in the abstracts.

**Fig 7:**
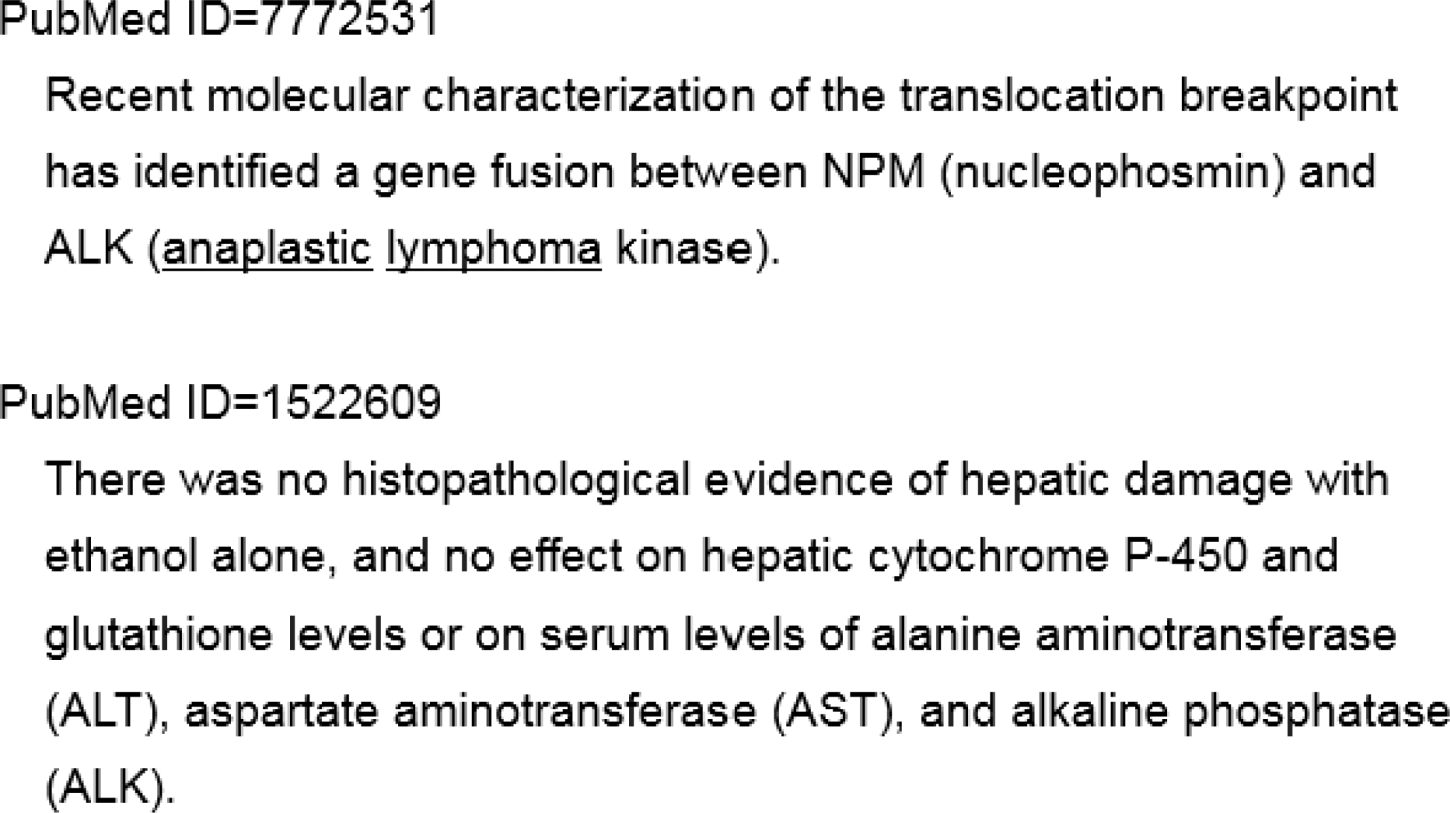
An example of a neighbor search. For the gene names of ALK, the following neighbor search words are generated: anaplastic, lymphoma, and CD246. In the first case (PubMed ID = 7772531), anaplastic and lymphoma occur. Therefore, this occurrence of ALK is considered to be positive. In the second case (PubMed ID = 1522609), none of these neighbor search words appear. Therefore, this occurrence is discarded.

Fig 7 shows an example of a neighbor search. Consider the case in which gene symbol ALK, Entrez Gene ID 238, occurs in an abstract. The gene names of ALK are anaplastic lymphoma receptor tyrosine kinase, CD246 antigen and mutant anaplastic lymphoma kinase. By splitting these gene names at the delimiters, we obtain a neighbor search word set: anaplastic, lymphoma, receptor, tyrosine, kinase, CD246, antigen and mutant. Among these words, receptor, tyrosine, kinase, antigen, and mutant are dropped as a neighbor search word set because they are not specific to ALK. Next, the neighbor search attempts to find any occurrence of one of these neighbor search words in the sentence in which ALK appears. In the first case, as shown in Fig 7 (PubMed ID = 7772531), we can see that the words anaplastic and lymphoma occur. On the other hand, in the second case (PubMed ID = 1522609), none of these words appear. Therefore, this occurrence of ALK is considered a false positive and is deleted from the gene occurrence table

Next, the occurrence table for aging-related terms was created by performing a keyword search, which consisted of PubMed IDs and the sentence numbers where the corresponding aging-related terms appeared in the abstracts. Then, we regarded the co-occurrence of genes and aging-related terms in a single sentence as an association between the two. In addition, the range of term co-occurrence varied, such as in one document, one paragraph, a single sentence, and a string of words of a fixed length. In general, a broad range generates high recall and low-precision results. We chose to use a single sentence in the same abstract to guarantee the accuracy of our results for this study. However, certain genes with the potential for classification as aging-related genes were not selected based on the aforementioned criteria. To guarantee quantity over quality, the co-occurrence of genes and aging-related terms in the same abstract was considered an association between the two.

Furthermore, to identify the highest number of aging-related genes, the pathway hypothesis was applied, which states that a gene is an aging-related gene if it occurs in the same pathway as a pre-established aging-related gene. First, the KEGG database [58], which provides information regarding molecular interactions, was checked for aging-related genes that co-occur in the same sentences as aging-related terms. Then, the genes that occur in the same pathway as aging-related genes were extracted and added to the list of aging-related genes.

### Classification and overview of integrated information

Detailed information related to aging-related genes, including the term co-occurrence frequency, was stored in our database. Because all of the data in the database were linked to gene IDs, a comprehensive aging-related catalogue on the axis of gene IDs could be created quickly. For example, the comprehensive catalogue may consist of identified aging-related genes, their related diseases, related foods, and the number of publications where the genes and other terms co-occur. By using data in the catalogue and the Heatplus package for R/Bioconductor, which offers several functions for producing a heatmap [32], aging-related genes can be classified and future trends in aging-related gene research can be predicted.

## Supporting information

S1 Table 4,227 aging-related genes identified based on the ‘single sentence’ criterion. (XLSX)

S2 Table 7,416 aging-related genes identified based on the ‘same abstract’ criterion. (XLSX)

S3 Table 128 genes co-occurring with all 14 terms. (XLSX)

S4 Table 68,260 gene symbols including synonyms. A zero in the Synonym column indicates a gene synonym, while one indicates a primary gene symbol.
(XLSX)
